# Length and PKA Dependence of Force Generation and Loaded Shortening in Porcine Cardiac Myocytes

**DOI:** 10.1155/2012/371415

**Published:** 2012-07-05

**Authors:** Kerry S. McDonald, Laurin M. Hanft, Timothy L. Domeier, Craig A. Emter

**Affiliations:** ^1^Department of Medical Pharmacology & Physiology, School of Medicine, University of Missouri, Columbia, MO 65212, USA; ^2^Department of Biomedical Sciences, College of Veterinary Medicine, University of Missouri, Columbia, MO 65212, USA

## Abstract

In healthy hearts, ventricular ejection is determined by three myofibrillar properties; force, force development rate, and rate of loaded shortening (i.e., power). The sarcomere length and PKA dependence of these mechanical properties were measured in porcine cardiac myocytes. Permeabilized myocytes were prepared from left ventricular free walls and myocyte preparations were calcium activated to yield ~50% maximal force after which isometric force was measured at varied sarcomere lengths. Porcine myocyte preparations exhibited two populations of length-tension relationships, one being shallower than the other. Moreover, myocytes with shallow length-tension relationships displayed steeper relationships following PKA. Sarcomere length-*K*_tr_ relationships also were measured and *K*_tr_ remained nearly constant over ~2.30 *μ* m to ~1.90 *μ* m and then increased at lengths below 1.90 *μ* m. Loaded-shortening and peak-normalized power output was similar at ~2.30 *μ* m and ~1.90 *μ* m even during activations with the same [Ca^2+^], implicating a myofibrillar mechanism that sustains myocyte power at lower preloads. PKA increased myocyte power and yielded greater shortening-induced cooperative deactivation in myocytes, which likely provides a myofibrillar mechanism to assist ventricular relaxation. Overall, the bimodal distribution of myocyte length-tension relationships and the PKA-mediated changes in myocyte length-tension and power are likely important modulators of Frank-Starling relationships in mammalian hearts.

## 1. Introduction

The primary role of cardiac myocytes is to develop force and power. In the isovolumic phase of the cardiac cycle, left ventricular myocytes develop (near)-isometric force against the enclosed ventricular chamber and in doing so increases ventricular pressure. When ventricular pressure exceeds aortic pressure the aortic valve opens, and myocyte force production is accompanied by loaded shortening (i.e., power) as blood is ejected from the ventricle into the systemic circulation. The rate of myocyte force generation determines the duration of isovolumic ventricular contraction and, consequently, the amount of the cardiac cycle devoted to ejection. The blood volume ejected per beat is determined by chamber compression, which is governed by (i) systolic ejection time, (ii) the number of force generating cross-bridges (which controls where on the force-velocity relation the ensemble of cross-bridges will work), and (iii) the inherent rate of loaded cross-bridge cycling. Hence, ventricular ejection is highly dependent on three myofibrillar mechanical properties: (1) force, (2) rate of force development, and (3) rate of loaded shortening (i.e., myocyte power output). There is considerable information related to these cardiac myofibrillar contractile properties in rodents [1–11], and these biophysical properties, in part, underlie the unique ventricular function of these species when compared to larger animals and humans [12–14]. However, there are fewer studies that have focused on cardiac myofibrillar mechanics in pig, a species of high translational relevance given its anatomic similarities to humans. Most investigations of porcine myofibrillar preparations have focused on steady-state properties at a single sarcomere length [15, 16] or examined stretch activation [17]. In this study we investigated three key myofibrillar mechanical properties (i.e., force, rate of force development, and loaded shortening) and their dependence on sarcomere length and PKA in porcine left ventricular cardiac myocytes. 

## 2. Methods

### 2.1. Animal Model

 Adult male yucatan miniature swine (14 months old) weighing 30–40 kg were obtained from the breeder (Sinclair Research Center; Columbia, MO). Animal care and use procedures complied with the *Guide* *for* *the* *Care* *and* *Use* *of* *Laboratory* *Animals* issued by the National Research Council and were approved by the University of Missouri Animal Care and Use Committee.

### 2.2. *In Vivo* Cardiovascular Function

 Animals (*n* = 4) were initially anesthetized with a telazol (5 mg/kg)/xylazine (2.25 mg/kg) mix and maintained using inhaled isoflurane (*≈*1.75%). Heparin was given with an initial loading dose of 300 U/kg IV, followed by maintenance of 100 U/kg each hour. A median sternotomy was performed and the pericardium was opened near the apex for insertion of the pressure-volume (*P*-*V*) loop catheter. *P*-*V* loops were measured utilizing a calibrated 7F admittance-based ADVantage catheter (SciSense; London, Ontario, Canada) positioned in the LV. A 14F balloon occlusion catheter was advanced to the inferior vena cava at the level of the apex of the heart via the deep femoral vein. Peripheral systemic MAP was measured via a fluid filled 6F LCB SH guide catheter (Boston Scientific) introduced through a 7F sheath placed in the right femoral artery and positioned in the descending aorta distal to the aortic band. Catheter placement was visualized and confirmed using angiography (InfiMed software) and Visipaque contrast medium. Following placement of the catheter, animals were brought to a peripheral MAP of 80 mmHg using phenylephrine (I.V. 1–3 *μ*g/kg/min) and allowed to stabilize for 5 minutes. *P*-*V* loops were collected before and after a single dose of dobutamine (5 *μ*g/kg/min I.V.) administered for 5 minutes under conditions of reducing preload achieved through transient occlusion of the inferior vena cava via inflation of the balloon catheter. Our admittance based *P*-*V* loop system requires input of baseline stroke volume (SV), which was determined one week prior to the terminal studies using ultrasound and calculated as previously reported [18] using the equation SV = *π*(*r*)^2^∗VTI where *r* is the radius and VTI is the velocity time interval (measured from apical four-chamber view). Aortic radius was calculated from the aortic left ventricular outflow track (measured in parasternal 2D view). 

### 2.3. Isolation of Cardiac Myocytes

 The heart was excised from the experimental animal following administration of a preanesthetic mixture of telazol (5 mg/kg)/xylazine (2.25 mg/kg) and permeabilized myocytes were isolated as previously described [19]. Briefly, a section of left ventricular free wall (~10 cm^3^) near the left anterior descending (LAD) coronary artery was removed and half was rapidly frozen in liquid nitrogen for biochemical analyses, and the other half was placed in ice cold relaxing solution for myocyte experiments. The piece in relaxing solution was cut into smaller pieces (2-3 mm) and homogenized with a Waring blender. The resultant slurry was centrifuged 75 sec at 165× g and the pellet was suspended for 3 min in 0.5% ultrapure Triton X-100 (Pierce Chemical Co.) in relaxing solution. The permeabilized myocytes were washed and centrifuged twice with cold relaxing solution with the final suspension kept on ice during the day of the experiment. 

For intact myocyte isolation, a section of the left-ventricular free wall was perfused via cannulation of the LAD. The tissue was perfused with a nominally calcium-free saline solution containing heparin for 10 minutes, followed by a minimal essential medium (MEM) solution containing 45 *μ*g/mL Liberase Blendzyme TH (Roche Applied Science, Indianapolis, IN, USA) for 30 minutes at 37°C. Digested tissue was minced and filtered, and the dissociated myocytes were washed and maintained in an MEM solution with 50 *μ*M calcium at room temperature until experimental procedures. 

### 2.4. Solutions

 Relaxing solution in which the ventricles were disrupted, skinned, and suspended contained (in mmol/L): EGTA 2, MgCl_2_ 5, ATP 4, imidazole 10, and KCl 100 at pH 7.0. Compositions of relaxing and activating solutions used in mechanical measurements were as follows (mmol/L): EGTA 7, MgCl_2_ 5, imidazole 20, ATP 4, creatine phosphate 14.5, pH 7.0, Ca^2+^ concentrations of 10^−9^ M (relaxing solution) and 10^−4.5^ M (maximal activating solution), and sufficient KCl to adjust ionic strength to 180 mM. The final concentrations of each metal, ligand, and metal-ligand complex were determined with the computer program of Fabiato [20]. Immediately preceding activations, muscle preparations were immersed for 60 s in a solution of reduced Ca^2+^-EGTA buffering capacity, identical to normal relaxing solution except that EGTA is reduced to 0.5 mM. This protocol resulted in more rapid steady-state force development and helped preserve the striation pattern during activation. Intact cardiomyocyte experiments were performed in a physiological saline solution containing (in mM). 135 NaCl, 4 KCl, 2 CaCl_2_, 1 MgCl_2_, 10 D-glucose, 10 Hepes, pH 7.4 with NaOH. 

### 2.5. Experimental Apparatus

The experimental apparatus for physiological measurements of myocyte preparations was similar to one previously described in detail [21] and modified for cardiac myocyte preparations [7]. Myocyte preparations were attached between a force transducer and torque motor by placing the ends of the myocyte preparation into stainless steel troughs (25 gauge). The ends of the myocyte preparations were secured by overlaying a 0.5 mm length of 3–0 monofilament nylon suture (Ethicon, Inc.) onto each end of the myocyte, and then tying the suture into the troughs with two loops of 10–0 monofilament (Ethicon, Inc). The attachment procedure was performed under a stereomicroscope (~100x magnification) using finely shaped forceps. 

Prior to mechanical measurements, the experimental apparatus was mounted on the stage of an inverted microscope (model IX-70, Olympus Instrument Co., Japan), which was placed upon a pneumatic vibration isolation table having a cut-off frequency of ~1 Hz. Mechanical measurements were performed using a capacitance-gauge transducer (Model 403-sensitivity of 20 mV/mg (plus a 10x amplifier) and resonant frequency of 600 Hz; Aurora Scientific, Inc., Aurora, ON, Canada). Length changes were introduced using a DC torque motor (model 308, Aurora Scientific, Inc.) driven by voltage commands from a personal computer via a 12-bit D/A converter (AT-MIO-16E-1, National Instruments Corp., Austin, TX, USA). Force and length signals were digitized at 1 kHz and stored on a personal computer using LabView for Windows (National Instruments Corp.). Sarcomere length was monitored simultaneous with force and length measurements using IonOptix SarcLen system (IonOptix, Milton, MA), which used a fast Fourier transform algorithm of the video image of the myocyte. Microscopy was done using a 40x objective (Olympus UWD 40) and a 2.5x intermediate lens. 

### 2.6. Sarcomere-Length Tension Measurements

 All mechanical measurements on cardiac myocytes were performed at 13 ± 1°C. For sarcomere length-tension measurements, an experimental protocol was performed similar to previously described [22]. Following attachment of myocyte preparation to the apparatus, the relaxed preparation was adjusted to a sarcomere length of ~2.35 *μ*m and then the preparation was maximally Ca^2+^ activated in pCa 4.5 solution. For sarcomere length-tension measurements, the cell preparation was transferred to a pCa solution that yielded ~50% maximal (i.e., pCa 4.5 or P_4.5_) force and then isometric force was measured over a range of sarcomere lengths monitored by the IonOptix SarcLen system (IonOptix, Milton, MA). Isometric force and sarcomere length were measured simultaneously. Sarcomere length was adjusted in a range between ~2.35 *μ*m and to ~1.4 *μ*m by manual manipulation of the length micrometer while the preparation was Ca^2+^ activated. After each sarcomere length change, ~10–15 seconds were provided to allow for development of steady-state force. Force at each sarcomere length was obtained via a slack-restretch maneuver (see below for description). For analysis, force at each sarcomere length was normalized to the force obtained at sarcomere length ~2.35 *μ*m (during the submaximal Ca^2+^ activation). Since force during submaximal Ca^2+^ activations invariably rose slightly during the sustained activation, normalized forces were calculated by interpolating force measurements at sarcomere length 2.35 *μ*m, which were performed at the beginning and end of the series of force measurements. At the end of each experiment, preparations were activated a second time in pCa 4.5 solutions and only experiments in which maximal tension remained >80% of initial were used for analysis. To assess the effects of PKA, length-tension relationships were performed before and after 45 min incubation with PKA (Sigma, 0.125 U/*μ*L). The pCa solution for length tension curves was adjusted to yield the same forces before and after PKA due to decreased Ca^2+^ sensitivity of force following PKA. 

### 2.7. Measurement of the Rate of Force Redevelopment, Loaded Shortening, and Power

 Force redevelopment rates were obtained using a procedure previously described for skinned cardiac myocyte preparations [23–25]. While in Ca^2+^ activating solution, the myocyte preparation was rapidly shortened by 15–20% of initial length (*L*_0_) to yield zero force. The myocyte preparation was then allowed to shorten for ~20 ms; after 20 ms the preparation was rapidly restretched to ~105% of its initial length (*L*_0_) for 2 ms and then returned to *L*_0_. Tension redevelopment following a slack-restretch maneuver was fit by a single exponential equation:

(1) F=Fmax⁡(1−e−ktr⁡t)+Fres,

where *F* is force at time *t*, *F*_max⁡_ is maximal force, *k*_tr⁡_ is the rate constant of force development, and *F*_res_ represents any residual tension immediately after the slack-restretch maneuver.

Power output of single skinned myocyte preparations was determined at varied loads as described earlier [26]. Briefly, myocytes were placed in activating solution and once steady-state force developed, a series of force clamps (less than steady-state force) were performed to determine isotonic shortening velocities. Using a servo-system, force was maintained constant for a designated period of time (200 to 250 msec) while the length change was continuously monitored. Following the force clamp, the myocyte preparation was slackened to reduce force to near zero to allow estimation of the relative load sustained during isotonic shortening; the myocyte was subsequently re-extended to its initial length. 

Myocyte preparation length traces during loaded shortening were fit to a single decaying exponential equation:

(2) L=Ae−kt+C,

where *L* is cell length at time *t*, *A*, and *C* are constants with dimensions of length, and *k* is the rate constant of shortening (*k*_shortening_). Velocity of shortening at any given time, *t*, was determined as the slope of the tangent to the fitted curve at that time point. In this study, velocities of shortening were calculated by extrapolation of the fitted curve to the onset of the force clamp (i.e., *t* = 0).

 Hyperbolic force-velocity curves were fit to the relative force-velocity data using the Hill equation [27]:

(3) (P+a)(V+b)=(P0+a)b,

where *P* is force during shortening at velocity *V*, *P*_0_ is the peak isometric force, and *a* and *b* are constants with dimensions of force and velocity, respectively. Power-load curves were obtained by multiplying force *x* velocity at each load on the force-velocity curve. The optimum force for mechanical power output (*F*_opt_) was calculated using [28]:

(4) Fopt=(a2+a∗P0)1/2−a.

Curve fitting was performed using a customized program written in Qbasic, as well as commercial software (Sigmaplot).

### 2.8. Intracellular Calcium Measurements

 Intact myocytes were plated on laminin coated coverslips and loaded with 5 *μ*M of the calcium indicator dye fluo-4/AM for 10 minutes, followed by a 20-minute wash. 2-dimensional laser-scanning confocal fluorescence microscopy was performed using the resonance scanhead of a Leica SP5 (Leica Microsystems, Buffalo Grove, IL, USA), with excitation at 488 nm and emission collected from 510–550 nm. Field stimulation (0.5 Hz) with a pair of platinum electrodes was used to induce action potentials and intracellular calcium transients. To analyze recovery kinetics, calcium transients were normalized using the following formula: [(*F* − *F*_baseline_)/(*F*_peak_ − *F*_baseline_)]. 

### 2.9. SDS-PAGE and Autoradiography

 The gel electrophoresis procedure was similar to one previously described [12, 29]. The gels for SDS-PAGE were prepared with a 3.5% acrylamide stacking gel and a 12% acrylamide resolving gel. Samples were separated by SDS-PAGE at constant voltage (250 V) for 8 h. Gels were initially fixed in a 10% acetic acid-50% ethanol solution, followed by 2% glutaraldehyde. MyHC isoforms were visualized by ultrasensitive silver staining, and gels were subsequently dried between mylar sheets.

PKA-induced phosphate incorporation into myofibrillar substrates was determined as described previously [30]. Briefly, skinned cardiac myocytes (10 *μ*g) were incubated with the catalytic subunit of PKA (5 *μ*g/mL) and 50 *μ*Ci [*γ*-^32^P] ATP at room temperature (21–23°C) for 45 minutes. The reaction was stopped by the addition of electrophoresis sample buffer and heating at 95°C for 3 minutes. The samples were then separated by SDS-PAGE for 2.5 hrs at 12 mA, silver stained, dried, and subsequently exposed to X-ray film for visualization. 

### 2.10. Statistics

 A mixed model incorporating linear regression and analysis of covariance was used to compare response variable (stroke volume) slopes plotted versus end diastolic volume, using treatment (baseline versus dobutamine) as the independent variable. Slopes of length-tension relationships were determined by linear regression. Paired *t* tests were used to determine whether there were significant differences in length-tension slopes and force-velocity parameters at two different sarcomere lengths or before and after PKA treatments. *P* < 0.05 was chosen as indicating significance. All values are expressed as means ± SD unless, otherwise, noted.

## 3. Results

### 3.1. Sarcomere Length Dependence of Force

**Figure 1:**
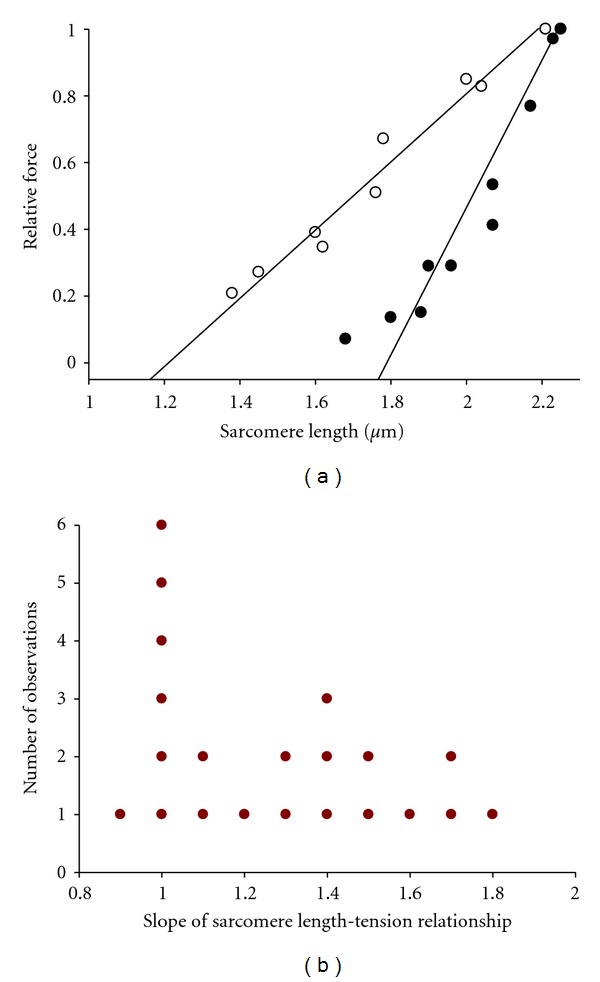
Sarcomere length-tension relationships in porcine skinned ventricular cardiac myocyte preparations. (a) Muscle cell preparations were mounted between a force transducer and motor, calcium activated to yield ~50% maximal force, then isometric force was measured over a range of sarcomere lengths monitored using an IonOptix SarLen system. (b) Histogram showing the slopes of length-tension relationships obtained in porcine cardiac myocytes.

**Figure 2:**
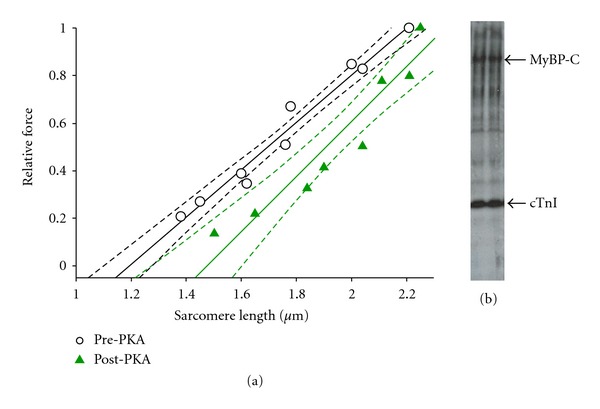
(a) Pig cardiac myocyte sarcomere length-tension relationships before and after PKA treatment. PKA-induced phosphorylation markedly steepened the length-tension relationship. (b) An autoradiogram showing radiolabeled phosphate incorporation into pig cardiac myofibrillar proteins (MyBP-C and cTnI) upon PKA treatment. Without PKA treatment, there was no radiolabelled ATP incorporation (data not shown).

 The characteristics of porcine left ventricular cardiac myocyte preparations are provided in Table 1. Steady-state sarcomere length-tension relationships were examined in myocyte preparations during near-half-maximal Ca^2+^ activations. Interestingly, porcine cardiac myocyte preparations exhibited a dichotomy of sarcomere length-tension relationships, some had shallow sarcomere length-tension relations while others displayed steep relationships (Figure 1). Histogram analysis of the length-tension relationship slopes indicates near bimodal distribution with one population of cells having a slope near 1.0 and another population with a slope near 1.5 (Figure 1), which has been similarly reported in rat and ferret myocyte preparations [22, 31]. We next examined whether PKA-induced phosphorylation of myofilament proteins may mediate the distribution of length-tension populations. PKA shifted a shallow length tension relationship to a steep length tension relationship implicating phosphorylation of myosin binding protein-C (MyBP-C) and/or cardiac troponin I (cTnI) as molecular modulators of sarcomere length-tension curves in porcine cardiac myocytes (Figure 2), as was previously observed in rat cardiac myocyte preparations [22].

### 3.2. Sarcomere Length Dependence of Rates of Force Development (*k*_tr⁡_)

**Figure 3:**
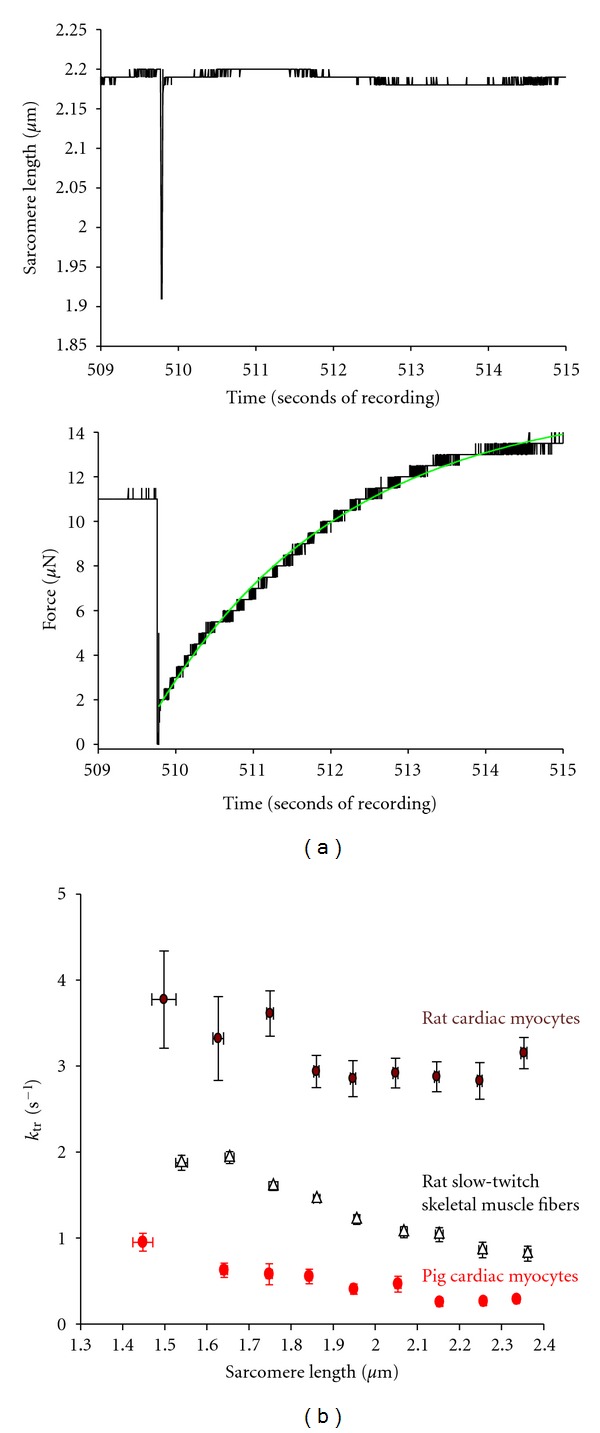
Sarcomere length dependence of the rate constant of force redevelopment (*k*_tr⁡_). (a) Slow time-based recordings of sarcomere length and force obtained using an IonOptix SarLen system during a slack restretch maneuver during submaximal Ca^2+^ activation. (b) Sarcomere length-dependence of *k*_tr⁡_ for rat slow-twitch skeletal muscle fibers, rat cardiac myocytes, and pig cardiac myocytes. Although pig cardiac myocyte *k*_tr⁡_ was much slower at all sarcomere lengths compared to rat cardiac myocytes, both pig and rat cardiac cell preparations showed that *k*_tr⁡_ increased at short sarcomere lengths despite reductions in force implicating that sarcomere length overrides the Ca^2+^ activation dependence of *k*_tr⁡_.

 The rate of force development is thought to mediate pressure development rates in mammalian ventricles. We examined the sarcomere length-dependence of force development rates in porcine cardiac myocytes. Force redevelopment was measured after a slack re-stretch maneuver, and the rate constant of force development (*k*_tr⁡_) was calculated by fitting a single concave exponential equation to the force trace. At sarcomere length ~2.30 *μ*m, *k*_tr⁡_ was ~0.3 s^−1^ during half-maximal activation, which was similar to previously reported for pig myocytes [32], nearly an order of magnitude lower than that measured in rat cardiac myocyte preparations, and only 30% of *k*_tr⁡_ values in rat slow-twitch skeletal muscle fibers, which like porcine cardiac myocytes contain the *β*-myosin heavy chain isoform. As sarcomere length was reduced from ~2.30 *μ*m to 1.90 *μ*m, *k*_tr⁡_ remained relatively constant, and then at sarcomere lengths below 1.90 *μ*m, *k*_tr⁡_ progressively increased. This *k*_tr⁡_-SL relationship was qualitatively similar to that observed in rat slow-twitch skeletal muscle fibers (Figure 3). Since force falls as sarcomere length is decreased but *k*_tr⁡_ increased with shorter sarcomere lengths, this implicates that sarcomere length **per * se* can override the well-described Ca^2+^-activated force dependence of rates of force redevelopment in cardiac muscle [33–35], that is, sarcomere length plays a dominant role in the kinetics of myofibrillar mechanical properties.

### 3.3. Sarcomere Length Dependence of Force-Velocity and Power-Load Curves 

**Figure 4:**
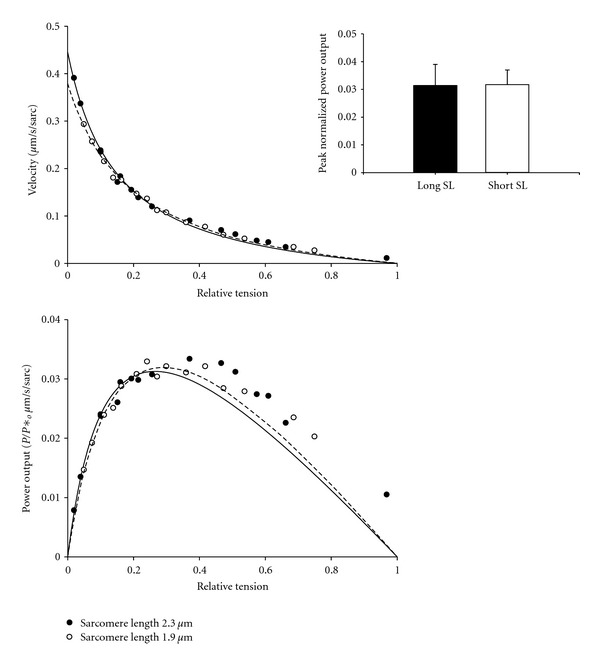
Normalized force-velocity and power-load curves from a pig left ventricular myocyte preparation at long and short sarcomere length obtained during half-maximal Ca^2+^ activations. Pig cardiac myocyte preparations exhibited little sarcomere length dependence of loaded shortening and power output. Inset shows bar plot (mean ± SD) of peak normalized power output at long (~2.30 *μ*m) and short (~1.90 *μ*m) sarcomere length (*n* = 8 myocyte preparations).

**Figure 5:**
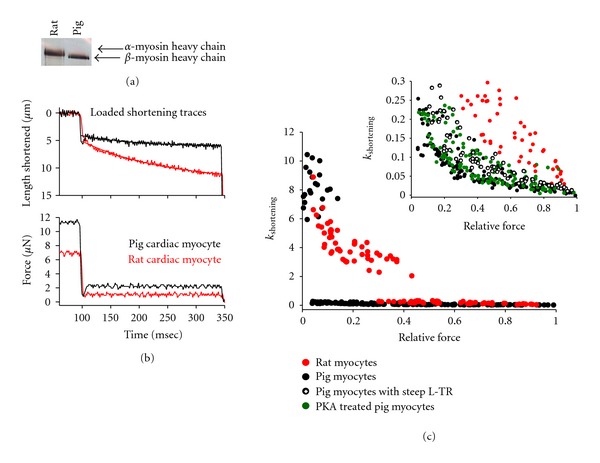
(a) Silver-stained gel showing the myosin heavy chain isoforms contained in a rat cardiac myocyte preparation compared to a pig cardiac myocyte preparation. (b) Representative length and force traces during a lightly loaded force clamp in a rat cardiac myocyte preparation (red) and a pig cardiac myocyte preparation (black) during a submaximal Ca^2+^ activation. (c) Length traces exhibited considerably greater curvature (greater *k*_shortening_) in rat myocytes compared to pig myocyte preparations at all relative loads. Inset in C shows an expanded plot of *k*_shortening_ versus relative load, which clarifies slope constants below 0.30. Interestingly, PKA-mediated phosphorylation increased the curvature (*k*_shortening_) of length traces towards those of rat myocyte preparations. In addition, pig cardiac myocyte preparations that exhibited steep length-tension relationships (L-T R) also had more curved length traces. This is consistent with PKA-mediated phosphorylation of myofilaments yielding greater responsiveness to changes in sarcomere length, in this case exhibited by greater shortening-induced cooperative deactivation.

Previous work has shown a tight regulation between isometric force and normalized force-velocity relationships in rat-skinned cardiac myocyte preparations [26]. However, in porcine cardiac myocyte preparations there was no force dependence of normalized force-velocity and power-load curves when force was altered by changing sarcomere length (i.e., force fell ~50% when sarcomere length was shortened from ~2.30 *μ*m to 1.90 *μ*m at the same submaximal activator [Ca^2+^], Figure 4). The finding that normalized myocyte power did not change over this sarcomere length range in pig myocytes differs from rat cardiac myocyte preparations where normalized force-velocity relationships were shifted downward at short sarcomere length (i.e., ~1.90 *μ*m versus ~2.30 *μ*m) at the same activator [Ca^2+^] [36]. The reason for this species difference is not known. One possibility is differences in cardiac myosin heavy chain; rat myocytes contain predominantly *α*-MyHC while pig myocytes contain mostly *β*-MyHC (Figure 5(a)). Interestingly, porcine *β*-MyHC has been shown to have a very slow actin-activated ATPase activity [37], which would prolong the duty cycle (i.e., cross-bridge cycle time spent strongly attached to actin). These strongly attached cross-bridges would tend to keep the thin filament activated [38–40] throughout the duration of the force clamp. This would sustain a relatively high number of cross-bridges to work against the load(s). This is consistent with linear length traces during load clamps in pig cardiac myocyte preparations (Figure 5(b)). The extent of curvature of length traces during load clamps is quantified by *k*_shortening_ values, which were much lower in pig myocytes than rat myocytes (Figure 5(c)). In pig myocytes, length traces were nearly linear (as indexed by *k*_shortening_ values) for most load clamps greater than 10% isometric force. This differs markedly from rat cardiac myocyte preparations, whereby length traces during force clamps deviate from linear at load clamps near 40% isometric force during submaximal Ca^2+^ activations (Figure 5(c)). 

### 3.4. PKA Effects on Loaded Shortening, Power Output, and *k*_shortening_

**Figure 6:**
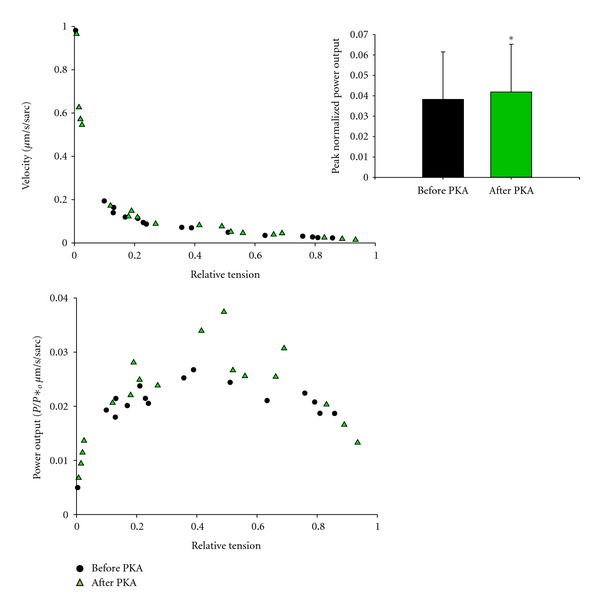
Normalized force-velocity and power-load curves from a pig left ventricular myocyte preparation before and after PKA treatment during half-maximal Ca^2+^ activations. Pig cardiac myocyte preparations exhibited more power after PKA treatment. Inset shows bar plot (mean ± SD) of peak normalized power output before and after PKA (*n* = 10 myocyte preparations).

**Figure 7:**
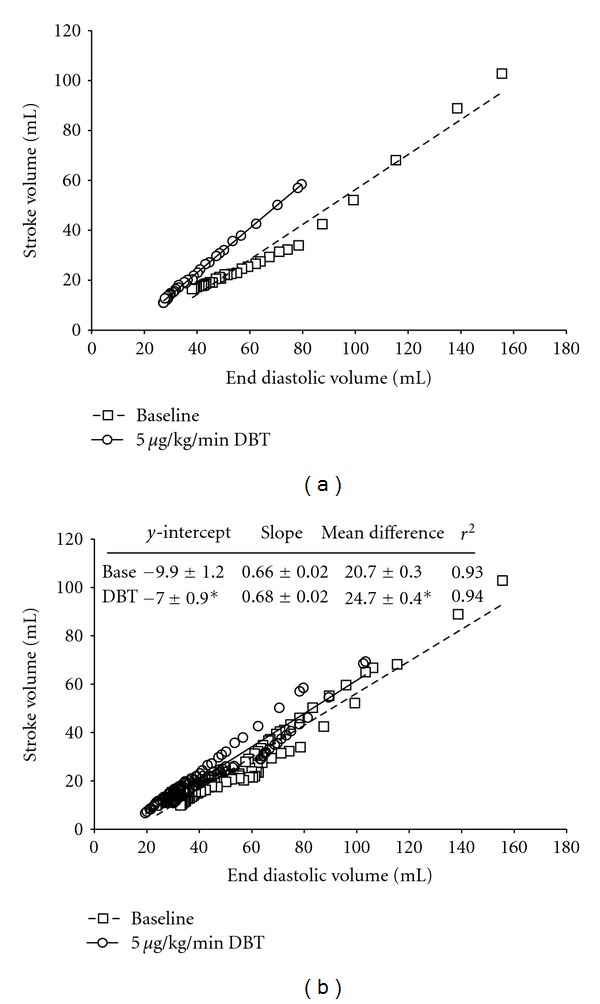
(a) Representative Frank-Starling relationship from one animal at baseline (Base) and after treatment with dobutamine (DBT). (b) Comprehensive group data from all animals illustrating a significant leftward shift in the Frank-Starling relationship (mixed model, treatment main effect adjusted for EDV covariance, *P* < 0.05). There was no significant interaction or change in slope of the Frank-Starling relationship between treatments (see table inset in (b)), therefore, parallelism was assumed. The *y*-intercept and marginal mean difference were both significantly increased following the dobutamine treatment (*P < 0.05; table inset (b)). The dobutamine challenge resulted in a ~15% increase in stroke volume (SV) for a given end diastolic volume (EDV) *in* *vivo*. This increase in ventricular function was similar in magnitude to that observed in our myocyte preparations (~10%), illustrating the coherence of our whole heart and cardiac myocyte functional data.

 We have previously shown that peak power generating capacity increases after PKA-mediated phosphorylation in rat cardiac myocyte preparations [25, 30] and such a change may contribute to augmented contractility in working hearts (i.e., more stroke volume at a given end-diastolic volume) [12]. We examined if a similar biophysical response would occur in pig cardiac myocyte preparations as a potential means for physiological changes in ventricular contractility in response to *β*-adrenergic stimulation and its downstream signaling molecule, protein kinase A (PKA). We observed a statistically significant increase in peak normalized power output after PKA treatment in pig myocyte preparations (Figure 6), however, the increase was considerably smaller than observed in rat myocyte preparations (a 10% increase in pig myocytes versus a 33% increase in rat myocytes [25, 30]). This small increase in myocyte power after PKA is consistent with a relatively small leftward shift in ventricular function illustrated by an ~15% increase in stroke volume for a given end diastolic volume that we observed in anesthetized pigs in response to a 5 *μ*g/kg/min dose of dobutamine at a mean arterial pressure of 80 mmHg (Figure 7).

Interestingly, PKA-mediated phosphorylation increased the curvature (*k*_shortening_) of length traces towards those of rat myocyte preparations (Figure 5(c)). In addition, pig cardiac myocyte preparations that exhibited steep length-tension relationships also had more curved length traces. This is consistent with the idea that PKA-mediated phosphorylation of myofilaments yields both greater force responsiveness to sarcomere length and greater shortening-induced cooperative deactivation.

In summary, pig cardiac myocyte preparations showed two populations of sarcomere length-tension relationships, which appear to be modulated by PKA. Sarcomere length overrode the Ca^2+^-activated-force dependence of *k*_tr⁡_ and loaded shortening. PKA treatment also slightly sped loaded shortening especially at loads optimal for power and yielded more curvilinear length traces during force clamps.

## 4. Discussion

In order to better understand the intricacies of heart function, it is necessary to determine the intermolecular control of myofibrillar contraction. In this study, we focused on three key myofibrillar functional properties (i) force, (ii) rate of force development, and (iii) power generating capacity, which together dictate ventricular stroke volume. We systematically examined these properties in porcine myofibrillar preparations. The study used pig ventricular myocardium for two main reasons: (1) pig hearts have many similarities to human hearts including heart size, heart rate, coronary circulation, responsiveness to many pharmacologic agents, and expression of mostly -myosin heavy chain (MyHC), and (2) to make comparisons with rat myocardium, which have been more extensively studied [7, 8, 12, 22, 25, 26, 30, 36, 41]. Overall, pigs likely provide an advantageous model to study cellular mechanisms of ventricular function and provide further basic insight into potential defects in cardiomyopathic states more related to the human condition. 

We observed that sarcomere-length dependence of force in pig myocyte preparations was very similar to that previously observed in rat cardiac myocyte preparations [22]. There was a dichotomy in the steepness of sarcomere length tension relationship whereby one population was shallower than the other. Interestingly, when myocyte preparations with a shallow length-tension relationship were treated with PKA, the relationships became steeper. While the exact molecular (posttranslational) modification by which PKA steepens length tension relationships remains to be determined, the finding is consistent with steeper ventricular function curves in response to *β*-adrenergic stimulation, assuming that myocyte length-tension contributes, at least in part, to the cellular basis of the Frank-Starling relationship. PKA also increased loaded shortening especially at loads near peak power and increased the curvature of length traces during force clamps. These PKA-mediated changes in myofibrillar function are consistent with physiological changes induced by *β*-adrenergic stimulation. *β*-adrenergic stimulation is known to (i) increase contractility (mediated in part by greater myocyte power at a given sarcomere length), (ii) steepen the Frank-Starling relationship (mediated in part by steeper length dependence of force), and (iii) speed relaxation (mediated, in part, by greater extent of shortening-induced cooperative deactivation manifested by more curved length traces). Interestingly, these myofibrillar changes in pig myocytes were all quantitatively less than those observed in rat cardiac myocytes, which is consistent with a slightly lower cardiac reserve that we have observed in pig hearts compared to rat and human hearts [42, 43]. 

**Figure 8:**
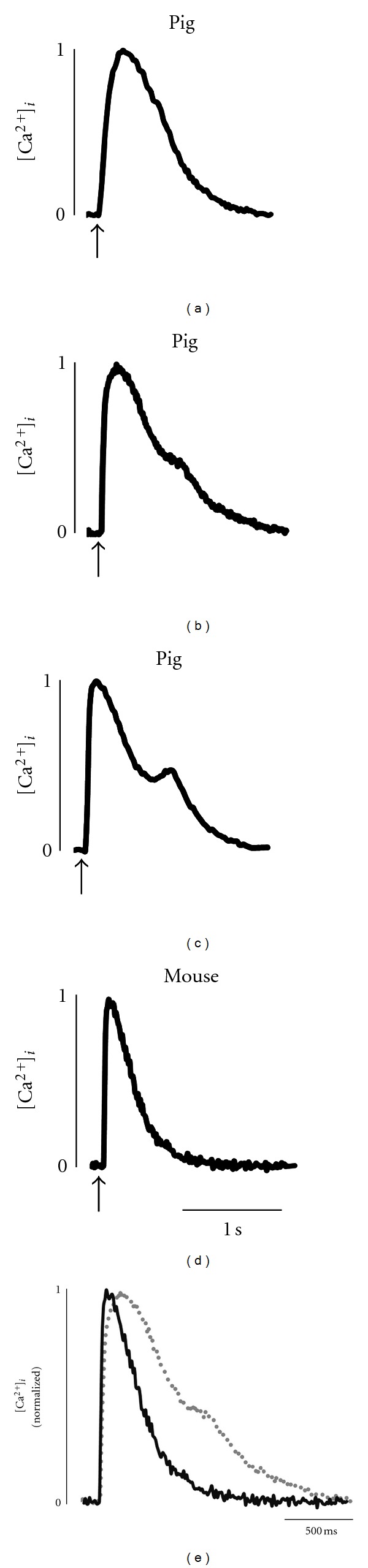
Representative amplitude-normalized calcium transients of Pig ((a)–(c)) and Mouse (d) left-ventricular myocytes (0.5 Hz, field stimulus denoted by arrow). Calcium transients from Pig exhibited multiple waveforms, including normal recovery from the transient peak (a, 2 of 14 cells), recovery with a marked shoulder ((b), 8 of 14 cells), and recovery with a secondary increase in calcium (c, 4 of 14 cells). (d) Mouse transients consistently exhibited a rapid transient recovery (*n* = 40). (e) Overlay of transients shown in (b) and (d) illustrating the distinct transient kinetics between pig (gray) and mouse (black) myocytes.

Additional myofibrillar mechanical properties observed in pig myocytes were that at the same activator [Ca^2+^] there was limited sarcomere length dependence of *k*_tr⁡_ and force-velocity relationships. The sarcomere length dependence of *k*_tr⁡_ was similar to that observed in rat slow-twitch skeletal muscle fibers in which *k*_tr⁡_ was similar over sarcomere length range of ~2.30 to 1.90 um and then increased at shorter sarcomere lengths. Since force falls over this entire sarcomere length range, this indicates that sarcomere length overrides the force dependence of *k*_tr⁡_ previously reported in cardiac muscle [33–35]. The mechanistic reasons for sarcomere length dominance of *k*_tr⁡_ is unclear but may indicate that cooperative activation of thin filaments is progressively reduced at shorter sarcomere lengths perhaps by more compliant thin filaments (i.e., shorter persistence length, which is the length that a mechanical force is transmitted along a functional entity), which would result in less recruitment of cross-bridges from the noncycling pool into the cycling pool, which has been proposed to limit rate of force development [35, 44]. Conversely, the lack of sarcomere length dependence of loaded shortening and power in pig myocytes differs from rat cardiac myocytes, where power decreased at short sarcomere length at the same activator [Ca^2+^] [36]. This may arise due to the very slow actin-activated ATPase activity reported for porcine *β*-MyHC [37]. Slow cross-bridge detachment would increase the population of strongly bound cross-bridges, which are thought to shift the thin filament equilibrium towards the open state by direct interaction with the actin-tropomyosin interface [40] and, at least in cardiac muscle, by increased affinity of cTnC for Ca^2+^ [45, 46]. Interestingly, we observed a marked shoulder in Ca^2+^ transients from intact pig myocytes (Figure 8). This shoulder was not observed in mouse myocytes that contain *α*-MyHC, which has a relatively short duty cycle. Mechanistically, the Ca^2+^ transient shoulder may arise from delayed Ca^2+^ dissociation from cTnC due to prolonged strongly bound attachment state(s) inherent to the long duty cycle of *β*-MyHC cross-bridges expressed in pig cardiac myocytes. Additionally, significantly delayed dissociation of Ca^2+^ from cTnC may elevate cytosolic Ca^2+^ at a time when ryanodine receptors have recovered from inactivation, yielding secondary Ca^2+^ induced-Ca^2+^ release from the sarcoplasmic reticulum. It does, however, appear that the long duty cycle of pig *β*-MyHC can be modulated by PKA mediated phosphorylation of myofibrillar proteins since PKA elevated power and yielded more curved length traces during load clamps (i.e., greater *k*_shortening_ values). These PKA mediated effects appear qualitatively similar across species and are thus likely to be of important physiological significance. In terms of ventricular contraction, PKA tends to increase the cooperative activation of thin filaments [47–49] providing more force-generating cross-bridges to work against an afterload yielding faster loaded shortening and, thus, more ejection. In terms of relaxation, the greater curvature of length traces implies greater shortening-induced cooperative deactivation, which would assist in ventricular relaxation to allow more time for filling and, thus, preserve stroke volume in the midst of higher heart rates. This mechanical signaling paradigm provides a myofibrillar mechanism to optimize cardiac output in response to high peripheral demands associated with stress (e.g., exercise).

## Figures and Tables

**Table 1:** Porcine cardiac myocyte preparation characteristics.

	*n*	Length (*μ*m)	Width (*μ*m)	Sarcomere length (*μ*m)	Passive force (*μ*N)	Maximum force (P_4.5_) (*μ*N)	Maximum force (P_4.5_) (kN m^−2^)
Cardiac myocytes	36	129 ± 28	28 ± 9	2.29 ± 0.06	0.70 ± 0.45	30 ± 14	75 ± 32

Values are means ± S.D.
